# Cardiorespiratory fitness in persons with lower limb amputation

**DOI:** 10.1097/MRR.0000000000000616

**Published:** 2024-02-26

**Authors:** Loeke van Schaik, Ilse J. Blokland, Klaske van Kammen, Han Houdijk, Jan H.B. Geertzen, Rienk Dekker

**Affiliations:** a University of Groningen, University Medical Center Groningen, Department of Rehabilitation Medicine, Groningen; b Department of Human Movement Sciences, Faculty of Behavioural and Movement Sciences, Vrije Universiteit Amsterdam, Amsterdam Movement Sciences, Amsterdam; c Heliomare Research and Development, Wijk aan Zee; d University of Groningen, University Medical Center Groningen, Center for Human Movement Sciences, Groningen, The Netherlands

**Keywords:** cardiopulmonary exercise test, cardiorespiratory fitness, lower limb amputation, physical fitness, rehabilitation

## Abstract

The aim of this study is to gain insight in the cardiorespiratory fitness of persons with lower limb amputation (LLA) during rehabilitation, and in potential factors influencing their cardiorespiratory fitness. We performed a retrospective cohort study using data from cardiopulmonary exercise tests. Included participants were adults with LLA. Main outcome was cardiorespiratory fitness expressed as $\dot{V}$O_2_ peak (ml/min/kg) and was directly determined using breath-by-breath gas analysis. $\dot{V}$O_2_ peak was compared to reference values for able-bodied controls. Multivariate regression analysis was performed to investigate potential factors related to $\dot{V}$O_2_ peak in persons with LLA. Potential factors were age, BMI adjusted, gender, level of amputation, aetiology of amputation, unilateral/bilateral, type of ergometry and use of beta blockers. Data of 74 participants with LLA are presented; 84% male (n = 62), mean age 58.9 (SD 11.6), mean BMI 26.7 (SD 5.6), 44 participants have a LLA above the knee, 30 below the knee. Overall $\dot{V}$O_2_ peak was lower in persons with LLA compared to reference values for able-bodied controls, with mean $\dot{V}$O_2_ peak for the total LLA group of 14.6 ± 4.1 ml/kg/min. In the multivariate regression analysis, only age was a significant predictor for lower $\dot{V}$O_2_ peak (regression coefficient: −0.15, 95% CI [0.23–0.069], r^2^ = 0.166). These results indicate that the cardiorespiratory fitness in persons with LLA is low, while they actually need more energy to walk and perform other daily activities. Cardiorespiratory fitness is not closely associated with the analysed demographic or clinical factors and will have to be determined on an individual basis for use in daily practice.

## Introduction

To better engage persons with lower limb amputation (LLA) in their rehabilitation process, it is important to have insight into their individual physical capabilities. In the Netherlands, there are different discharge destinations for persons after LLA, namely home, nursing home or rehabilitation center [1]. This choice is made, among other things, on the basis of the pre-existent level of physical functioning. In general, older people with LLA, who have often been inactive for a long time, go to a nursing home for rehabilitation. The persons who were more mobile before LLA usually undergo clinical or outpatient rehabilitation in a rehabilitation center. It is of particular interest to evaluate the individual physical capabilities of persons with LLA who are referred to a rehabilitation center as there is evidence that persons with LLA who have higher levels of physical fitness are more likely to gain ability to walk with a prosthesis [2–4]. Insight in the maximum cardiorespiratory fitness (CRF) can therefore be helpful to identify persons with below-average physical fitness and compose better rehabilitation programs to train and improve CRF. Improved CRF can enable persons with LLA to reach their individual rehabilitation goals, improve walking abilities, and improve independence and quality of life [2,5–7]. Knowledge about individual CRF can also be used to discuss, guide and manage expectations from the perspective of both the person with LLA and the professional [8–10].

Cardiopulmonary exercise tests (CPETs) are used to reliably determine a person’s maximum CRF, expressed as maximum oxygen uptake ($\dot{V}$O_2_ peak). In the general population, reference values for $\dot{V}$O_2_ peak are available, classified by gender and age [11–13]. It is known that the CRF decreases with increasing age and that men have higher levels of CRF compared to women [14]. In persons with LLA, limited studies are available on CRF. The reported CRF in persons with LLA is lower compared to those of able-bodied controls [14–18]. It should be noted that in some of these studies, the CRF is not directly measured using breath-by-breath gas analysis, but an estimation is made based on submaximal tests or heart rate and therefore have limited reliability [18,19].

Different types of ergometers are used in LLA studies; arm-crank ergometer, one leg ergometer or combined arm-leg ergometer [17,20–22]. In able-bodied individuals lower cardiorespiratory values are reported using arm-crack ergometry vs. leg ergometry, due to the limited muscle mass engaged in arm ergometry which causes reduced stress on the cardiovascular system [23]. However, in persons with LLA, a recent study compared $\dot{V}$O_2_ peak measured with arm-crank ergometer vs. one leg ergometry and found no difference in $\dot{V}$O_2_ peak values [21]. This outcome can probably be explained by the fact that the muscle mass of one leg is equal to that of two arms. Furthermore, lower values are reported for one leg ergometry vs. two leg ergometry in persons with LLA [22]. These findings suggest that both ergometers can be used to assess $\dot{V}$O_2_ peak in persons with LLA, but research is needed to confirm that the type of ergometer does not influence the measured $\dot{V}$O_2_ peak values.

As mentioned above, only a few studies reported on CRF in persons with LLA. The main limitations of these studies are the small sample size and the inclusion of participants with LLA due to nonvascular reasons [17,21]. Because the majority of persons have a LLA due to peripheral vascular disease, these results are not representative of the entire population of persons with LLA [24]. In general, persons with cardiovascular comorbidities are more likely to have lower CRF [15,25]. Therefore, it is hypothesized that the CRF values in the vascular LLA group are even lower compared to the reported values in persons with LLA due to nonvascular reasons. Given the comorbidity and deconditioning in persons with vascular LLA, it is even more important to gain knowledge about their CRF.

Therefore, the aim of this study is to provide insight in the CRF in persons with LLA during rehabilitation, regardless of the level or cause for LLA. Additionally, the aim is to analyze which factors (age, BMI adjusted, gender, level of amputation, aetiology of amputation, unilateral/bilateral, type of ergometry and use of beta blockers may predict the level of CRF.

## Methods

### Participants

This study included adults (≥18 years) with LLA who were referred for CPET as part of their rehabilitation process at the Rehabilitation Center of Heliomare between January 2018 and November 2022. For this retrospective study, data was used from the CPET-database of Heliomare. The inclusion of the participants was according to the American College of Sports Medicine criteria [26]. The CPET-database was reviewed by the medical ethical committee of Vrije Universiteit Medical Centre Amsterdam and approved by the local ethical committee of Heliomare. Informed consent was gained from the participants with LLA. Participants were selected based on ICD codes. When the same participant performed more than one CPET, for example, for test-retest purpose, data of the first CPET was selected.

### Data collection

The following descriptive data was collected from the database: age, gender, BMI, amputation level, aetiology of amputation, date of amputation, use of beta-blockers and type of ergometry. BMI was determined using the adjusted body weight, calculated as described previously [27,28].

### Cardiopulmonary exercise test measurements

The CPET was performed with an arm ergometer or a cycle ergometer [22], under supervision of a certified clinical exercise physiologist and/or physician. The protocol depended on the type of ergometer. For the arm ergometer, it was a ramp protocol, for the one-legged ergometer a block protocol. After a 3-minute rest and 3-minute warm-up (0 Watt), the physician determined the protocol based on the estimated maximum CRF, as previously described [18,29]. The test ended if the participant could not maintain a cycle pace >50 revolutions per minute, or if the physician had another reason to stop. Afterwards, 3-minute cooling down followed. Breath-by-breath gas exchange, ECG and oxygen saturation were continuously monitored.

Specific outcomes of the CPET recorded in the database include peak oxygen uptake ($\dot{V}$O_2_ peak) (ml/kg/min) (the maximum of the 30 s averaged $\dot{V}$O_2_ was considered $\dot{V}$O_2_ peak), peak respiratory exchange ratio (RER) during exercise phase of the test, peak heart rate (HR) (beats/min), and peak power (Watts).

$\dot{V}$O_2_ peak is the highest level of oxygen uptake attained during the CPET for that person, regardless of reason for test termination. Note that although other literature may refer to this variable as $\dot{V}$O_2_ max [16,30–32], in persons with LLA (but also in other patient groups) it is possible the maximum effort is limited due to other reasons than the capacity for oxygen uptake, for example, balance problems or musculoskeletal pain. Hence for this study, the term ‘ $\dot{V}$O_2_ peak‘ is used.

The $\dot{V}$O_2_ peak-value was considered valid when the participant reached a RER > 1.1 or HR > 85% predicted maximal HR. This conforms to daily practice following the American College of Sports Medicine criteria [26].

### Statistical analysis

Statistical analyses were performed using IBM SPSS Statistics 28. P-values <0.05 were considered significant.

Participant characteristics were presented with mean and SD, median and range or frequencies and percentages in case of nominal data.

The CPET outcomes $\dot{V}$O_2_ peak (ml/kg/min), RER peak, peak HR (beats/min), and peak power (Watts) were presented with mean and SD.

Individual $\dot{V}$O_2_ peak values were presented based on age and gender. Because in the general population, CRF depends on age and gender, we continue to use this subdivision in the figures.

### Multivariate regression analysis

Multivariate regression analysis was performed to predict $\dot{V}$O_2_ peak in participants with LLA. Factors that were analysed as potential predictors were age, BMI adjusted, gender, level of amputation, aetiology of amputation, unilateral/bilateral, type of ergometry and use of beta blockers. The variable ‘aetiology of amputation’ was categorized in two groups: vascular/diabetes (DM) vs. other (e.g. trauma/oncological/infection). The level of amputation was categorized in two groups: below the knee (BK) (Syme or transtibial) vs. levels above the knee (AK) (knee disarticulation, transfemoral or hip disarticulation).

First, an exploratory analysis was performed to determine which independent variables were associated with $\dot{V}$O_2_ peak. Because on group level the $\dot{V}$O_2_ peak was not normally distributed, non-parametric testing (Mann–Whitney U-test or Spearman Rho) was used. All factors showing an association, as defined by *P* < 0.20, were selected for further analysis.

Subsequently, if the association between two factors was biologically plausible, we performed a Pearson Chi-Square test of association and, if significant, avoid the collinearity between the two factors by only entering the variable with the strongest association in the regression analysis.

Finally, to determine predictive value, the selected factors were entered into a multivariate linear regression analysis following a backward stepwise procedure. Factors with the highest p-values were removed until all remaining factors were significant (*P* ≤ 0.05). Given the non-normal distribution of $\dot{V}$O_2_ peak, the regression analysis was checked with a bootstrapping procedure. In case of no difference, the original confidence intervals were presented. Otherwise, robust estimates were presented.

## Results

Data from 79 participants were available. One participant performed the CPET from a wheelchair and was excluded. CPET results of four individuals were excluded for not reaching maximal effort (RER < 1.1 and predicted HR < 85%), which was due to musculoskeletal pain. None of the participants had to stop the CPET due to cardiovascular events. No obvious ECG abnormalities or drops in blood pressure were seen. Descriptive statistics are presented in Table 1. Comorbidity is based on reporting in the referral letter or CPET form.

**Table 1:** Descriptive characteristics participants

		Mean (SD) or number [% (range)]
Gender (male)^a^		62 (84%)
Age (years)^a^		58.9 (11.6) (range 25–79)
BMI^a^*		26.7 (5.6) (range 17.1–43.3)
Level of amputation^a^	Below knee amputation Above knee amputation	44 (59.5%) 30 (40.5%)
Aetiology of amputation^b^	Vascular/DM Other totals - Trauma - Oncology - Infection - Other	47 (63.5%) 24 (32.4%) 6 5 7 6
Unilateral vs. bilateral^a^	Unilateral Bilateral	69 (93.2%) 5 (6.8%)
Type of ergomete^a^	Arm ergometer One leg cycle ergometer	45 (61%) 29 (39%)
Comorbidity^b^	Vascular disease Diabetes Hypertension COPD Polyneuropathy Cerebrovascular accident	44 23 10 4 3 3
Use of beta blockers^c^	Yes No	17 (23.0%) 51 (68.9%)
Time since amputation in months^a^^,^^d^		1.4 (0.4–452.0)^e^

*BMI was calculated with the adjusted weight value [27].

a n = 74;

b n = 71;

c n = 68;

d At the time of the cardiopulmonary exercise test (CPET).

e Median (range) instead of mean (SD).

### Cardiopulmonary exercise test

**Fig. 1:**
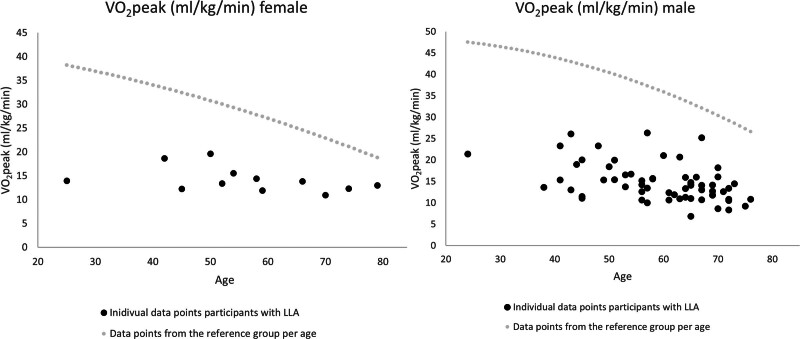
(a) $\dot{V}$O_2_ peak in females; individual data points participants and reference line able bodied. $\dot{V}$O_2_ peak in females; individual data points participants and reference line able-bodied [13]. (b) $\dot{V}$O_2_ peak in males; individual data points participants and reference line able bodied. $\dot{V}$O_2_ peak in males, the individual data points participants and reference line able-bodied [13].

Descriptive CPET data for the 74 participants with LLA are presented in Table 2. The mean $\dot{V}$O_2_ peak was 14.6 ± 4.1 mL/kg/min. In Fig. 1a and b, the individual $\dot{V}$O_2_ peak data points for female and male participants respectively are presented as a function of age along with the reference based on data for able-bodied controls in the Dutch/Flemish population [13]. In male participants mean $\dot{V}$O_2_ peak was 14.6 (±4.4) ml/kg/min, with a mean age of 59.5 (±11.6) years. In the age-matched reference group, males had a reported $\dot{V}$O_2_ max of 38.5 (±9.0) [13]. Female participants had a mean $\dot{V}$O_2_ peak of 14.1 (±2.6) ml/kg/min, with a mean age of 56.2 (±10.9). In age-matched reference group, the reported mean $\dot{V}$O_2_ max was 29.5 (±7.8) [13].

**Table 2:** Descriptive results of the cardiorespiratory exercise test

	Mean ± SD	Range
$\dot{V}$O_2_ peak (ml/kg/min)^a^	14.6 ± 4.1	6.8–26.3
RER peak^a^	1.2 ± 0.1	1.03–1.59
Peak heart rate (beats/min)^b^	137.5 ± 27.2	80.0–194.0
Peak heart rate (%pred)^b^	0.87 ± 0.2	0.54–1.27
Peak power (watts)^a^	71.1 ± 31.7	18.0–170.0

a n = 74.

b n = 73.

### Multivariate linear regression

Test-values and p-values per factor are presented in Table 3. Factors used in the multivariate linear regression analysis were age, BMI adjusted, aetiology of amputation and use of beta-blockers (*P* < 0.20). Because of the potential association between aetiology of amputation and the use of beta blockers, a Pearson Chi-square analysis was performed but it was NS (χ^2^ = 2.920, *P* = 0.087). Hence both factors were included in the regression analysis.

**Table 3:** The test-values and *P*-values for the possible predictors for $\dot{V}$O_2_ peak

	Test-values	*P*-value
Age	−0.465^a^	<0.001*
BMI adjusted	−0.192^a^	0.101**
Gender	389.0^b^	0.943
Unilateral vs. bilateral	154^b^	0.622
Aetiology of amputation	780^b^	0.024*
Level of amputation	806^b^	0.218
Use of beta-blockers	590^b^	0.056**
Kind of ergometry	691^b^	0.992

a Spearman rho.

b Mann–Whitney-U test.

*P < 0.05, **P < 0.20.

The final model (Table 4) indicates that the only significant predictor was age, showing that increased age is associated with lower predicted $\dot{V}$O_2_ peak. The regression coefficient for age was -0.15, meaning that with every 10 year increment in age, $\dot{V}$O_2_ peak decreased by 1.5 mL/kg/min. The explained variance of the final model was 17% (average r^2^ = 0.166).

**Table 4:** Multivariate linear regression analysis results

Predictor	Regression coefficient	Std. error	*P*-value	95% confidence interval	
				Lower bound	Upper bound
Intercept	23.32	2.34	< 0.001	18.64	28.00
Age	−0.15	0.04	< 0.001	−0.23	−0.069

## Discussion

This study aimed to determine the peak CRF ($\dot{V}$O_2_ peak) of persons with LLA during clinical outpatient rehabilitation in a representative center in the Netherlands. Our results showed that in general, the $\dot{V}$O_2_ peak in the included participants with LLA is lower compared to reference values for the able-bodied population, and that age was the only significant predictor for $\dot{V}$O_2_ peak in the included participants with LLA.

Our results in both male and female participants (Table 2) were more than 50% lower than the respective age-matched normative values [12,13]. Overall, they were also lower on average than the previously reported $\dot{V}$O_2_ peak values in persons with non-vascular LLA of 28.1 (6.7) ml/kg/min [18,19,33] and close to the values reported for vascular LLA of 17.1 (4.1) ml/kg/min [19]. Despite the heterogeneity in participant characteristics, there is no single participant in this study exceeded the reference line of able-bodied controls (Fig. 1a and b), possibly due to deconditioning, comorbidities and changed body composition with the loss of (a part of) the leg.

Additionally, previous studies report that walking with a prosthesis requires more energy than walking for able-bodied [34]. The combination of higher energy costs in walking and the reduced CRF makes persons with LLA use a much larger part of their physical capacity which influences self-selected walking speed but also affect participation and quality of life [34,35].

The multiple regression analysis only revealed age as a significant predictor for CRF. In our sample, older age was correlated with lower CRF, which is also seen in the able-bodied population [14]. In the able-bodied population, males show higher $\dot{V}$O_2_ max values compared to females [13]. In our sample, however, gender was not found as a significant predictor for $\dot{V}$O_2_ peak. This finding can be explained by the limited number of female participants(n = 12). Moreover, the hypothesis was that the $\dot{V}$O_2_ peak values for the vascular LLA group would be lower compared to the LLA group with other causes. We did not find this, probably due to the heterogeneity in participant characteristics (e.g. level of LLA) and the small sample size, with a relatively small number of non-vascular LLA. The explained variance of the final model was 17%, which supports the suggestion that due to the small sizes of the subgroups, the data are too heterogeneous to obtain a well-fitted model.

In the general LLA population in the Netherlands, mean age is 74.2 years and more than 90% of the LLA is due to vascular/DM reasons [24,36]. Therefore, although there were no specific inclusion criteria, this study sample is not quite representative of the general LLA population in the Netherlands. This is probably due to the fact the CPETs took place in a rehabilitation centre meaning there is a selection bias because in the Netherlands not everyone with LLA is referred to a rehabilitation centre. The majority of elderly with LLA, often with more comorbidities, are admitted to a nursing home for their rehabilitation [1] as mentioned before in the introduction. Possibly the current data is still an overestimate of $\dot{V}$O_2_ peak in the entire population of individuals with LLA.

In this study, participants were tested on an arm ergometer or on a one-leg cycle ergometer, group sizes respectively 44 and 30. Our results showed no significant association between type of ergometry and $\dot{V}$O_2_ peak (Table 3). Given these findings, both types of ergometer could be considered appropriate for determining the $\dot{V}$O_2_ in individuals with LLA. In able-bodied, there is a difference in $\dot{V}$O_2_ peak values depending on type of ergometer [23]. However, our findings are consistent with previous findings using arm ergometry vs. one leg ergometry in persons with LLA [21] and values reported in persons after knee surgery [37]. The CPET ergometer can be selected according to the capabilities or preference of individuals with LLA.

All participants were able to perform the CPET without complications. Despite the variety of comorbidities, none of the participants had to stop the test due to any serious (cardiorespiratory) events. Only four participants did not reach the required RER or >85% predictive HR for a valid test, all due to musculoskeletal pain. Therefore, when following the American College of Sports Medicine inclusion guidelines, a CPET can be safely performed in persons with LLA regardless of cardiovascular comorbidities.

### Strength and limitations

To our knowledge, this is the largest study that examined CRF in persons with LLA using breath-by-breath gas analysis during CPET. Although some CPET data used here were previously reported [18], this was done to increase the sample size for examining several plausible predictors. Although 74 participants is a decent sample size in amputation research, the main difficulty remains the heterogeneity. To analyse specific predictors such as gender or aetiology of LLA, which are theoretically likely predictors for CRF, a larger sample size for these subgroups is required. Another strength is that in this study, $\dot{V}$O_2_ peak was directly determined using breath-by-breath gas analysis during a CPET. This is in contrast to some other studies in which CRF was predicted based on HR or submaximal testing [20]. Furthermore, as mentioned before, the measurements took place in a rehabilitation centre, therefore the reported $\dot{V}$O_2_ peak is not necessarily generalizable for the total LLA population.

### Conclusion

CRF ($\dot{V}$O_2_ peak)in participants with LLA in a rehabilitation setting (clinical or outpatient) is low compared to the reference values reported for age- and gender-matched able-bodied controls. Higher age is associated with lower $\dot{V}$O_2_ peak in persons with LLA. To gain more insight into other potential predictors and establish reference values for persons with LLA, more data is needed to be able to compare larger subgroups. CPET for determining CRF in persons with LLA is feasible and it would be recommended to include CPET as a standard test during rehabilitation in order to evaluate individual CRF to better organize the rehabilitation program with regard to fitness training.

## Acknowledgements

We would like to thank the Heliomare rehabilitation centre and the participants for their contribution.

### Conflicts of interest

There are no conflicts of interest.
